# MASCoD—Multidimensional Assessment of Subjective Cognitive Decline

**DOI:** 10.3389/fpsyg.2022.921062

**Published:** 2022-11-30

**Authors:** Marina Maffoni, Antonia Pierobon, Cira Fundarò

**Affiliations:** ^1^Psychology Unit, Istituti Clinici Scientifici Maugeri IRCCS, Montescano Institute, Montescano, Italy; ^2^Neurophysiopatology Unit, Istituti Clinici Scientifici Maugeri IRCCS, Montescano Institute, Montescano, Italy

**Keywords:** subjective cognitive decline, subjective memory complaint, subclinical cognitive impairment, Mild Cognitive Impairment, dementia, multidimensional assessment, neuropsychology

## Abstract

Subjective cognitive decline (SCD) is a subclinical cognitive impairment that is complained by the individual without being objectively supported at clinical, diagnostic, and neuropsychological levels. It can negatively impact on patient’s frailty and quality of life, as well as on the caregiver’s burden. Moreover, it can be prodromal to Mild Cognitive Impairment or dementia. Although the clinical manifestations of SCD can differ along with several cognitive domains, to date there are only screening tools to investigate subjective memory complaints. Thus, the first aim of this paper is to propose a preliminary English and Italian version of a new screening tool called MASCoD (Multidimensional Assessment of Subjective Cognitive Decline); the second aim is to propose its preliminary adoption on a pilot sample. This schedule is a brief test derived from the review of the literature and the clinical experience provided by an experts panelist. From pilot tests, it seems promising as it can help the professional to make differential diagnosis and to predict the risk of developing severe cognitive impairment over time, developing a personalized care path. This screening tool is brief, easily embeddable in usual clinical assessment, and administrable by different professionals. Furthermore, following validation, it will allow to collect manifold cognitive manifestations of SCD, addressing the shortage of previous validated instruments globally assessing cognition affected by this condition.

## Introduction

The population aging increases the attention on possible manifestations of pathological cognitive decline. Some forms of cognitive impairment can be insidious and not immediately evaluable with neuropsychological tests or other clinical measures. In this case, it is possible to speak about subjective cognitive complaints–SCC (Jacob et al., 2019; Numbers et al., 2021), better known as subjective cognitive decline–SCD (Jessen et al., 2014; Molinuevo et al., 2017; Jessen et al., 2020) as defined by an international working group of researchers and clinicians (Jessen et al., 2014).

Even if the literature itself is unclear about the real nature of SCD, from a phenomenological perspective, SCD refers to perceive, in daily living, a decline in some areas of cognition without objective proofs of cognitive impairment through formal neuropsychological and instrumental evaluations (Studart and Nitrini, 2016; Jessen et al., 2020). The individuals who suffer from SCD commonly complain of memory problems, mental slowness and concentration difficulties, and self-perceived deterioration in usual cognitive performance. Among these symptoms, the most reported problem concerns memory as the individual reports difficulties in remembering names and recent events or appointments (Mitchell, 2008; Studart and Nitrini, 2016). Consequently, as far as we know, researchers and clinicians have exclusively developed tests that assess possible memory complaints; between the most recent there are Prospective and Retrospective Memory Questionnaire—PRMQ (Smith et al., 2000; Sala, 2020); Memory Complaint Scale–MCS (Vale et al., 2012), Kihon Checklist-Cognitive Function–KCL-CF (Tomata et al., 2017). A less recent but well-known and used instrument is called “Memory Complaint Questionnaire–MAC-Q” (Crook et al., 1992). It is a rapid self-report questionnaire constituted by six items scored on a 5-point Likert scale which asks the individual to compare her/his actual ability in memory tasks with the performance of the past (Crook et al., 1992; Reid et al., 2012). Although it is still used in research, it presents several limitations, mainly linked to the influence of the individual’s affective status and other factors not linked with memory, such as executive functioning and age-related psychomotor speed (Reid et al., 2012). Moreover, it does not consider other manifestations of SCD, for example, the subjective experience of being disoriented or overwhelmed when asked to make decisions or plans. Moreover, psycho-physical frailty is not considered as MAC-Q was developed when this construct was still in its infancy and, in some ways, it is still a subject of debate (Proietti and Cesari, 2020). However, the logic and structure of MAC-Q can still offer useful hints for detecting the subjective memory complain.

Concerning the epidemiologic perspective, a consistency between studies is still lacking (Röhr et al., 2020; Si et al., 2020). However, some studies reported a prevalence of SCD as high as 50–60% among community-dwelling older adults (Holmen et al., 2013; Singh-Manoux et al., 2014). In addition, a recent study combining data for 16 cohorts from 15 countries has shown that the prevalence of SCD largely varied across studies (from 6.1 to 52.7%), overall reaching roughly one in four older adults without a diagnosis of cognitive impairment (Röhr et al., 2020). Moreover, this subclinical cognitive disorder was higher in men, in people with lower levels of education and living in lower- and middle-income countries, in Asian and Black African people, as well as in research conducted in later decades (Röhr et al., 2020). Interestingly, being female, and having a lower educational level and low socioeconomic status are not only associated with SCD, but also with frailty, which is a multidimensional syndrome resulting in increased psychosocial and physical vulnerability in older adults (Margioti et al., 2020). Regarding this, SCD is significantly associated with various frailty indexes and, overall, it can be considered an effective frailty indicator in the cognitively unimpaired older population (Hsieh et al., 2018; Gifford et al., 2019; Margioti et al., 2020).

Over time, the trajectories of SCD can be three: (a) reversible complaint due to other etiologies (e.g., mood disorders, medication side-effects), (b) non-reversible but stable complaint probably due to physiological aging, (c) progressive cognitive decline (Jessen et al., 2020; Liew, 2020b). Specifically, SCD can be a prodromal symptom of Mild Cognitive Impairment—MCI (van Harten et al., 2018; Boel et al., 2022) or dementia (Liew, 2020a; Numbers et al., 2021; Topiwala et al., 2021). Concerning this, SCD can be associated with Alzheimer’s biomarkers such as amyloid plaques and tau proteins (Jack et al., 2013; Amariglio et al., 2015; Sierra-Rio et al., 2016). Moreover, a meta-analysis unveiled that 25% of cognitively healthy older people who complain SCD develop MCI in the following 4 years and have a twofold risk of progressing to dementia in the subsequent 5 years (Mitchell et al., 2014). Coherently, an increasing number of studies report that SCD can easily result in MCI and, in turn, in Alzheimer’s disease or other kinds of cognitive decline (Mendonça et al., 2016; Studart and Nitrini, 2016; Molinuevo et al., 2017; Lin et al., 2019; Numbers et al., 2021; Topiwala et al., 2021), particularly when it is associated to anxious and depressive manifestations (van Rijsbergen et al., 2019; Liew, 2020a).

Moreover, it is worth to be said that cognitive difficulties characterizing SCD may negatively impact the individual’s health-related quality of life, daily activities and may ignite anxious and/or depressive conditions (Pusswald et al., 2015; Roehr et al., 2017). Furthermore, SCD can be associated with the caregiver’s burden (Sheikh et al., 2018).

Keeping in mind these considerations, the early detection of SCD is of paramount importance in order to make differential diagnosis and, in case of persistent cognitive impairment, to track its progressive development. Thus, a rapid screening test usable in clinical practice which assesses all nuances of the SCD has to be considered urgent and imperative in the current healthcare landscape to effectively support the individual’s and caregiver’s health status. As far as our knowledge, there is not yet a multidimensional screening tool investigating not only the subjective memory complaint, but also other risk factors, as well as cognitive and emotional manifestations of SCD.

In this vein, this paper firstly aims to propose a preliminary version of a novel screening test for healthcare professionals to detect early symptoms of SCD and to follow the patients over time, tailoring a proper diagnostic and supportive care path. Moreover, a secondary aim is to propose preliminary data on a pilot clinical adoption of the MASCoD to assess its feasibility.

## Methods

### Instrument Development

The schedule development involves the psychologists and neuropsychologists of the Psychology Unit and neurologists of the Neurophysiopathology Unit, employed at ICS Maugeri of Montescano, a clinical and research hospital located in the North of Italy. Indeed, this paper mainly describes the conceptualization and construction of the instrument. On a voluntary basis, a panel of 10 experts in cognitive functioning and disorders (neurologists, psychologists, neuropsychologists) has been constituted, and several planned meetings have been scheduled to discuss SCD. Then, participants agreed on aims, individual tasks, and they established a project timeline for the future research protocol.

Specifically, after preliminary scheduled online and in-person group sessions, MM and CF reviewed the literature, searching for previously validated screening instruments and collecting the updated knowledge on SCD. Then, all authors discussed the findings and started the conceptualization of the instrument based on the literature review and their extensive clinical experience. In particular, they chose a user-friendly model to define the cognitive processes involved when responding to a questionnaire despite being a healthcare professional or a patient: question interpretation, retrieval of relevant information from memory and context, judgment formulation, choice of response options on the bases of previous judgment and question understanding (Sudman et al., 1996). In line with this model, we formulated the items of each cognitive domain potentially affected by SCD. Moreover, potential biases affecting items presentations and responses have been considered and addressed by the authors (Van Herk et al., 2004).

When suitable, original or slightly modified items from other validated items were selected, previously asking authors’ written permission or specifying the original references. Overall, all items constituting the new screening have been discussed among the authors and they were repeatedly revised until the full consensus was reached concerning both the content and the language, in order to refine the reliability and clinical usefulness of the new instrument.

The items selected in this preliminary version of MASCoD came from suggestions “from the field” that can be a winning strategy for reaching an effective and easy schedule in clinical practice. An example is provided by the Psychosocial Cardiological Schedule that is currently usable in cardiorespiratory rehabilitation units (PCS; Pierobon et al., 2012; Granata et al., 2020).

We unveiled also some preliminary cut-offs by dividing the maximum total score (21) by three and, then, tuning the three levels as follows: low risk = 0–7; medium risk = 8–13; high risk = 14–21. This stratification has a clinical purpose as detecting different risk levels of developing cognitive issues over time might allow to propose tailored intervention, maximizing the outcome and optimizing resources.

Finally, the authors provided a collaborative and iterative translation of the schedule in the English language as suggested in the literature to preserve the ecological value (Douglas and Craig, 2007).

The name chosen for the new screening tool is the following: MASCoD—Multidimensional Assessment of Subjective Cognitive Decline.

### Participants to Preliminary Administration and Assessment of Clinical Feasibility

To evaluate the clinical feasibility, a preliminary adoption of this screening has been carried on with some outpatients during the neurological interviews within the DTCP of the hospital (Table 1): A trainee psychologist compiled the items according to the answers provided by the patients along with the usual anamnestic, clinical and diagnostic neurological assessment.

**TABLE 1:** Socio-demographic characteristics of patients (*n* = 26).

Variable	*n*	%
**Gender**		
Female-male	18–8	69.23–30.77
**Age**		
≤49	2	7.69
50–60	3	11.54
61–70	5	19.23
71–80	8	30.77
≥81	8	30.77
**Education**		
None	0	–
Primary school	6	23.09
Middle school	12	46.15
High school	4	15.38
Bachelor degree	0	–
Master degree	4	15.38
**Current occupation**		
Craftsman	0	–
Staying in parent	1	3.85
Unemployed	0	–
Manager	1	3.85
Employee	1	3.85
Businessman	0	–
Teacher	1	3.85
Freelancer	1	3.85
Worker	1	3.85
Disable	1	3.85
Retired	15	57.67
Other	4	15.38
**Whom do you live with**		
By myself	7	26.83
Husband/wife/partner	12	46.25
Son/daughter	2	7.69
Partner and children	5	19.23
Parents	0	–
Others (no family members)	0	–
**Primary caregiver**		
Husband/wife/partner	9	34.62
Son/daughter	9	34.62
Other members, not family (caregiver)	1	3.85
Other person (friends)	4	15.38
Nobody	3	11.53
**Actual smoking**		
Yes	3	11.53
No	16	61.55
Yes, in the past	7	26.83
**No. of comorbidities/other risk factors**		
0	6	23.08
1	13	50
2	4	15.38
3	2	7.69
≥4	1	3.85

Afterward, MASCoD results have been compared, in a contingency table, to the usual neuropsychological evaluation included in the DTCP of the hospital, following an effective approach already used in previous research (Pierobon et al., 2018). Although SCD is not observable through neuropsychological assessment, previously it has been highlighted some areas of weakness in the cognitive profile of people reporting this condition, related mainly to memory, and attention or executive functioning (López-Higes et al., 2018).

MASCoD is going to be validated and tested in clinical practice. Specifically, the recruitment will address all eligible inpatients of ICS Maugeri IRCCS—Institute of Montescano (PV), and outpatients who will undergo the dementia Diagnostic-Therapeutic Care Pathway (DTCP) of the same hospital.

### Ethical Considerations

Principles of transparency and scientific rigor are adopted in order to develop a new screening tool grounded in clinical experience and updated literature knowledge, maximizing strengths and minimizing possible drawbacks (Guberman et al., 2001; Iragorri and Spackman, 2018; Kishore et al., 2021). Moreover, this instrument has been developed as a side project of a broader research protocol assessing the impact of cognitive impairment on the patient-caregiver dyad coping with cognitive decline. Among these, some patients and/or caregivers complain SCD, not yet ascribable to an objective cognitive impairment (DYAD4DEM, CEC N.2315, 11/06/2019; Torlaschi et al., 2021).

The current study was reviewed and approved by the Technical and Scientific Committee of the ICS Maugeri Institute of Montescano (PV) on 21st April 2022 (MON03/22) and by the Ethical Committee of the Maugeri Institute, too (protocol number CE 2666, 26 July 2022).

## Results

### Multidimensional Assessment of Subjective Cognitive Decline Description

The first assessment and follow-up versions of this instrument are composed of a general form for socio-demographic data and three sections which assess the main risk factors and cognitive domains related to SCD. It can be filled by the neurologists, neuropsychologists, or other healthcare professionals who want to investigate the patient’s reported SCD.

The complete instrument in the English and Italian languages and the scoring rules can be retrieved in the Supplementary Material.

### Clinical and Socio-Demographic Data

A general form for collecting the main individual’s socio-demographic and clinical data is proposed. This information can be easily retrieved from the usual anamnestic interview conducted by the healthcare professionals during the assessment. This general form refers to the patient’s personal information (i.e., name, surname, education, marital status, and working activity), her/his social support, and non-specific health risk factors (Body Mass Index, tobacco use, substance abuse, cardiological pathologies, etc.).

### Section A—Risk Factors

The first section of the screening assesses the characteristics of SCD which increase the risk of developing cognitive decline according to literature (Jessen et al., 2014, 2020). There are items asking for the patient’s age, the onset of SCD, subjective worries, and confirmation of cognitive complaint by an external informant. Moreover, the presence of concomitant neurological and/or cognitive pathologies or further relevant comorbidities is also investigated.

In the follow-up version, the healthcare professional has to consider the risks factors unveiled in the first assessment (1–10 scores) and add possible cerebral/neurological diseases or other comorbidities that occurred after the last assessment.

### Section B—Subjective Cognitive Manifestations

This section aims to collect all possible manifestations of SCD in daily living. After written consent, we adopted a slightly modified version of “MAC-Q,” one of the most used screenings for subjective memory complaints (Crook et al., 1992). The amendments update the items and make them more ecological for the current generation (e.g., “Recalling *telephone numbers or zip codes* that you use on a daily or weekly basis?” has been replaced with “Recalling *password or other access codes* that you use on a daily or weekly basis?”). Moreover, the 5-points Likert scale has been turned into a yes/no answer in order to both be consistent with the test and provide a more rapid evaluation.

Aiming to assess other possible features of SCD than memory complaints, we propose five additional items concerning attention or concentration problems, as well as the experience of being disoriented or overwhelmed by decision-making or daily task duties. For instance, the patient is asked for moments of black-out or attention failure, and possible difficulties in following conversations or planning.

Overall, all items are focused on the subjective feeling of deterioration in performance and greater difficulty in completing tasks than in the past. The focus is to pinpoint subjective cognitive manifestations in daily life.

### Section C—Psychological Manifestations

The last section does not contribute to the total score (0–21), rather it is thought to help the professional to make differential diagnosis and correctly define the risk level and, in turn, to tailor the diagnostic and clinical care path. Specifically, a screening for anxious and depressive symptoms is proposed as they may be misinterpreted as cognitive issues or may worsen the cognitive impairment (van Rijsbergen et al., 2019; Jessen et al., 2020; Liew, 2020a). To screen anxiety and/or depressive symptoms have been chosen the recently Italian validation of GAD-2 (Generalized Anxiety Disorder; Spitzer et al., 2006; Kroenke et al., 2007; Giuliani et al., 2021) and PHQ-2 (Patient Health Questionnaire; Spitzer et al., 1999; Kroenke et al., 2003; Giuliani et al., 2021), as they are the shortened versions of two well-established and very used screening tools for mood disorders in research and clinical practice (Giuliani et al., 2021).

Furthermore, a question on stressful life events that occurred in the last year has been proposed. Although it does not contribute to the score of section C because of the ceiling effect, this information can help the professional to make differential diagnosis between possible mood disorders due to distressing experiences or cognitive impairment. Stressful life events can be negative (e.g., bereavement, fired at work or retirement, end of a relationship) or positive (e.g., marriage, birth, career passage) experiences that result in a relevant amount of distress for the individual. A checklist of the main negative or positive stressful life events is provided in the literature (Holmes and Rahe, 1967).

### Scoring Procedure

Each item requires a dichotomous response. Only in Section A, it is required to also add an open-ended explanation to declare possible comorbidities.

To assess the severity of the SCD condition, it is assigned a score of one point if a risk factor is present. Thus, summing the scores of section A and section B, it is possible to define the individual risk of developing MCI or dementia over time. Specifically, dividing the maximum total score by three, we propose some preliminary cut-offs as follows: low risk = 0–7; medium risk = 8–13; high risk = 14–21. These risk levels are warning flags that guide the healthcare professional to choose the appropriate clinical and diagnostic strategy. The current cut-offs can be considered preliminary cut-offs and deserve to be recalibrated following a well-defined validation study.

In section C, the scoring over or under the clinical cut-off for depressive/anxious symptoms (Giuliani et al., 2021) and the occurrence of positive/negative relevant stressful events (Holmes and Rahe, 1967) are moderators which can help the healthcare professional to understand the unveiled profile and to better determine the most effective care path for the individual. Indeed, in case of severe emotional problems, a tailored psychological supportive intervention can be primarily considered, as cognitive manifestations can be the misleading epiphenomenon of psychological issues.

Overall, this scoring method has been considered the most suitable for a screening tool as the results are intuitive and easily comprehensible.

### Clinical Feasibility and Preliminary Descriptive Data

From a qualitative perspective, the feasibility of the current version of the instrument is quite satisfying concerning clinical usefulness and administration time.

To now, 26 MASCoD were compiled by the trainee psychologist, retrieving the patients’ information during the baseline neurological assessment related to the DTCP of the hospital. The main socio-demographic characteristics of this sample were shown in Table 1.

According to clinical judgment and DTCP guidelines, 13 of these patients were also provided with a screening or a comprehensive neuropsychological assessment. Afterward the risk indexes unveiled by MASCoD and neuropsychological assessment were qualitatively compared for investigating the levels of data convergence. Specifically, MASCoD scores of 1 (low risk) or 2 (medium risk with depressive/anxious symptoms) highlight a profile not so cognitively impaired (1) or suggest a psychological explanation for subjective symptoms (2). On the contrary, MASCoD scores of 3 (medium risk without depressive/anxious symptoms) or 4 (high risk) highlight a more impaired cognitive profile. Although these cut-offs need to be better analyzed from a psychometric perspective, unveiling different risk levels is pivotal for this new schedule that aims to tailor follow-up and interventions, early detecting individuals with higher risk to develop cognitive impairment. Focusing on the screening or comprehensive neuropsychological assessment, a severe (deficits in two or more cognitive domains) or slightly (only one deficit in cognitive domains) impaired score were considered suggestive of cognitive impairment (MCI or dementia). Thus, comparing SCD and neuropsychological assessment, the convergence level (true positive and true negative) is 69.24% and the divergence level (false positive and false negative) is 30.76% (Table 2).

**TABLE 2:** Contingency table comparing SCD and neuropsychological assessment (*n* = 13).

	MCI or dementia YES	MCI or dementia NO
	***n* (%)**	***n* (%)**

High level of SCD (MASCoD scores: 3–4)	3 (23.08) True positive	2 (15.38) False positive
Low level of SCD (MASCoD scores: 1–2)	2 (15.38) False negative	6 (46.16) True negative

*MCI or dementia YES, only one or many deficits in two or more cognitive domains; MCI or dementia NO, no deficits in cognitive domains.*

## Discussion

Assessing the SCD on its onset has to be considered pivotal in the present and future healthcare scenario in order to early detect a progressive cognitive impairment or make differential diagnosis of mood disorders or non-stable complaints derived from other organic causes (Jessen et al., 2020; Liew, 2020a; Numbers et al., 2021). However, as far as we know, a multidimensional screening test for the assessment of manifold manifestations of SCD is still lacking in the international literature. Specifically, there are various questionnaires assessing memory complaints, such as MAC-Q (Crook et al., 1992), but they do not address other possible features of SCD related to frailty, attention or executive abilities, as well as possible comorbidities with anxious and depressive symptoms.

In this vein, the present paper proposes a new screening tool for assessing SCD in clinical practice which is going to be validated. As secondary aim, we propose some preliminary data concerning its clinical feasibility. Following a validation study that is currently under consideration, MASCoD might suggest to the healthcare professional a tailored diagnostic and clinical path to follow the patient on the bases of the risk to develop a severe cognitive impairment over time. Specifically, this instrument will enable to describe the SCD at low, medium, or high risk to turn into MCI or dementia according to clinical and scientific evidences.

Addressing the first aim, it has to be said that MASCoD is composed by three different sections. Specifically, *Section A* allows detecting features increasing the risk that SCD results in MCI or dementia over time. In this regard, the literature showed that the onset of SCD before the age of 60 years old, the persistence of SCD over time, worries and confirmation of this complaint by an external informant, and the presence of other comorbidities can be warning signs of preclinical forms of cognitive decline, increasing the likelihood to develop MCI or dementia in the following years (Jessen et al., 2014, 2020; Numbers et al., 2021; Topiwala et al., 2021). Thus, it is relevant to check the presence of these risk factors to predict the most likely trajectory of SCD for each patient.

*Section B* focuses on phenomenological expressions of SCD in the everyday life. According to literature describing memory complaints as the main symptom of SCD (Jessen et al., 2020; Numbers et al., 2021), the majority of items concern tasks requiring the use of memory, such as recalling names, plans, or access codes. We adopted the instrument called MAC-Q (Crook et al., 1992) as it is the most used screening for memory complaints related to SCD and, after the consent, we slightly modified the items in accordance with generational changes as suggested in the literature (Reid et al., 2012). We attempted to bridge the gap between literature and clinical practice, proposing some items concerning attention or concentration problems, and the self-perception of being disoriented or overwhelmed by decision-making or daily task duties. As far as we know, this is the first instrument that aims to assess possible failure in global cognition and the on-going validation study will assess its construct validity and clinical usability. Indeed, literature and clinical practice report that manifestations of SCD can fluctuate and change from patient to patient (Si et al., 2020). Thus, it is important to investigate not only memory complaints but also other features of SCD to better understand the prognosis of this condition.

Furthermore, *Section C* provides rapid screening of anxious and/or depressive symptoms through an *ad hoc* question on stressful life events and two well-known instruments, GAD-2 (Spitzer et al., 2006; Kroenke et al., 2007; Giuliani et al., 2021) and PHQ-2 (Spitzer et al., 1999; Kroenke et al., 2003; Giuliani et al., 2021). This is a response to the necessity to investigate the etiology of subjective complaints because, at least in some cases, some impairments can be due to reversible causes, such as affective disorders or drug side effects (Jessen et al., 2020; Liew, 2020a; Si et al., 2020). Thus, it is necessary to make an adequate differential diagnosis in order to differentiate and tailor the diagnostic care path. Specifically, in case of suspicion of anxiety and/or depressive conditions, it is crucial to propose an effective diagnostic and supportive intervention at a psychological and psychotherapeutic level. If no other concurrent organic or cognitive causes exist, psychological interventions over time can be effective in reducing and/or resolving the manifestations of SCD and alleviate the caregivers’ burden (Sheikh et al., 2018).

Concerning the second aim of this paper, preliminary data showed a satisfactory convergence level of MASCoD scores and usual neuropsychological assessment. Specifically, compared to usual neuropsychological assessment, MASCoD seems to be a promising tool for correctly detecting the most probable trajectory of developing cognitive decline over time. A bigger sample will deepen and strengthen these preliminary findings through a well-structured validation study that is going to start. To now, this comparison through a contingency table can be considered only from a clinical perspective in order to gain hints to further test the scale.

Overall, the multidimensional assessment offered by MASCoD can be suitable in the current and future healthcare scenarios to address the increasing attention to SCD unveiled during clinical assessment. The awareness concerning the cognitive domain is increased in society, so professionals are asked to early detect clinical or preclinical cognitive symptoms, implementing preventive and supportive interventions tailored to every single patient. Indeed, this is a precondition to manage the possible consequences of cognitive impairment on frailty (Hsieh et al., 2018; Margioti et al., 2020), medication adherence (Maffoni and Giardini, 2017; Giardini et al., 2018; Maffoni et al., 2020, 2021) and caregivers’ burden (Sheikh et al., 2018; Torlaschi et al., 2021). Moreover, early detection and understanding of SCD grant the possibility to propose effective intervention for counterattacking the progression of this condition, managing its consequences, and orienting psychoeducational and neuropsychological interventions (Ranzini et al., 2020; Roheger et al., 2021).

In this vein, following validation, this new screening tool might be a useful schedule thanks to the fact that it is brief, easily embeddable in usual clinical assessment, and administrable by different professionals. Furthermore, it aims to collect manifold cognitive manifestations of SCD directly from the patient’s perspective, addressing the shortage of previously validated instruments globally assessing cognition.

However, MASCoD has to be considered in light of its limits. Firstly, the version presented in this paper is preliminary and a well-structured research project has been approved by the Scientifical Ethical Committee and it will be soon presented to the Ethical Committee of our Institute in order to start the validation study as soon as possible. Secondly, items and cut-offs predicting low, medium, or high risk that SCD can result in MCI or dementia are derived through a consensus of the experts’ panel. Thus, longitudinal studies and the use of this instrument during the clinical practice will allow redefining and better tuning of cut-offs on the bases of evidence-based knowledge and validation studies. Specifically, well-known quantitative techniques deserve to be implemented to select the items of the final version of the instrument, as well as to assess psychometric characteristics and validity and reliability of cut-offs. Finally, further international research is welcomed to both test the validity and reliability of this tool also in other cultures and to explore its usability in other clinical settings other than neurological assessment. For instance, it would be interesting to investigate the administration of MASCoD by family general practitioners or directly by the patients themselves. Indeed, this test aims to monitor the trajectory of the SCD over time and, in turn, to address the choice of screening and/or in-depth neuropsychological assessment.

## Conclusion

This new screening schedule MASCoD can become a useful instrument for carrying out a systematic active search of risk factors, memory and non-memory manifestations of SCD in everyday life, as well as for making differential diagnosis with anxiety/depressive symptoms or disorders linked to other organic causes. As this screening involves dichotomous questions and it is administrable to various healthcare professionals, it could become in the future an effective tool to quickly assess SCD and predict its most likely trajectory. Thus, it might support the clinical practice, helping the professional to propose a tailored diagnostic and clinical taking care of the patient over time.

Although MASCoD deserves further psychometric and clinical explorations, we consider important to present this schedule to the scientific community in order to collect suggestions and hints which can pave the way to the subsequent validation phase of the tool. Putting together different perspectives is, indeed, pivotal in order to gain an effective schedule for the identification and definition of SCD which plays a relevant role in the early diagnosis of neurodegenerative cognitive disorders.

## Data Availability Statement

The preliminary data of this study are available from the corresponding author upon reasonable request.

## Ethics Statement

The studies involving human participants were reviewed and approved by the Technical and Scientific Committee of the ICS Maugeri, Institute of Montescano, and Ethical Committee of the Maugeri Institute (protocol number CE 2666). Written informed consent for participation was not required for this study in accordance with the national legislation and the institutional requirements.

## Author Contributions

MM, CF, and AP made substantial contributions to the conception and construction of the screening tool. MM wrote the first draft of the manuscript. AP and CF provided important suggestions to the content and reviewed the manuscript. All authors have read and agreed to the current version of the manuscript.

## Conflict of Interest

The authors declare that the research was conducted in the absence of any commercial or financial relationships that could be construed as a potential conflict of interest.

## Publisher’s Note

All claims expressed in this article are solely those of the authors and do not necessarily represent those of their affiliated organizations, or those of the publisher, the editors and the reviewers. Any product that may be evaluated in this article, or claim that may be made by its manufacturer, is not guaranteed or endorsed by the publisher.
